# Development and Validation of the Defending Behavior Scale of Cyberbullying for Adolescents

**DOI:** 10.3390/bs14100967

**Published:** 2024-10-18

**Authors:** Hong Chen, Chuan Chen, Yunduan Li, Cuiying Fan

**Affiliations:** 1 College of General Education, Chongqing Vocational Institute of Engineering, Chongqing 402260, China; 2 School of Chinese Language and Literature, Central China Normal University, Wuhan 430079, China; chenchuan9303@mails.ccnu.edu.cn; 3 School of Psychology, Northwest Normal University, Lanzhou 730070, China; 2021104105@nwnu.edu.cn; 4 Key Laboratory of Adolescent Cyberpsychology and Behavior (CCNU), Ministry of Education, Wuhan 430079, China; 5 School of Psychology, Central China Normal University, Wuhan 430079, China

**Keywords:** cyberbullying, defending behavior, adolescents, reliability, validity

## Abstract

In the context of cyberbullying, bystanders engage in a diverse array of defending behaviors, employing a range of strategies tailored to the specific forms of bullying they encounter. These defending behaviors have been demonstrated to effectively mitigate the adverse effects of cyberbullying on victims. This study involved the development of the defending behavior scale of cyberbullying for adolescents and a subsequent assessment of the scale’s reliability and validity. Firstly, the structure of defending behavior was explored based on the results of in-depth interviews and previous studies in the literature. The defending behavior scale of cyberbullying for adolescents was then developed, and 550 middle school students were selected for item analysis and exploratory factor analysis. Secondly, 526 middle school students were selected for a series of reliability and validity analyses, including tests of convergent validity, discriminant validity, and structural validity, to assess the reliability and validity of the scale. The final version of the scale comprises 24 items distributed across four dimensions: emotional support, reporting authority, aggressive defending, and problem-solving. The four factors collectively account for 66.26% of the total variance. The internal consistency coefficient α of the scale was 0.92, with the internal consistency coefficients α of the dimensions being 0.88, 0.92, 0.92, 0.92, and 0.87, respectively. The scale demonstrated good convergent validity, discriminant validity, structural validity, and criterion validity. Finally, the scale has measurement equivalence across genders. The defending behavior scale of cyberbullying for adolescents was demonstrated to possess good reliability and validity, to meet the requirements of psychometrics, and to be suitable for use in measuring defending behavior in adolescent cyberbullying.

## 1. Introduction

Cyberbullying is an aggressive behavior in which an individual or group of individuals uses electronic information exchange tools to repeatedly and persistently victimize individuals who cannot easily defend themselves [1]. Cyberbullying is quite common in all countries or regions of the world, and its incidence is increasing year by year, and the groups in which it occurs are mostly adolescents [2]. Approximately 14.6% to 56.9% of adolescents have been exposed to various forms of cyberbullying, such as being verbally abused, harassed, denigrated, and having their privacy exposed [3]. In earlier studies, researchers categorized the roles in cyberbullying into four types, namely, cyber bully, cyber victim, bully victim, and bystander [4]. This division views bystanders as outsiders in cyberbullying who have no direct relationship with the bullying incident. This may be because most bystanders choose to remain silent during cyberbullying. Bystanders in cyberbullying are individuals who witness the cyberbullying incident [5]. Since bystanders witness the bullying incident, they observe and perceive the existence of bullying behavior. As a result, bystanders react differently; for example, they can choose whether to get involved or ignore it [6].

In cyberbullying incidents, positive bystander behaviors can provide various types of help to victims [7], for example, stopping the bullying incident, reducing the impact of the bullying incident [8], or reporting it to an authority figure (e.g., a teacher), which allows the bullying incident to be dealt with effectively [9]. Such positive behavior in cyberbullying is also known as defending behavior. Defending behavior is the prosocial behavior of any bystander who tries to prevent a bullying incident from worsening or escalating [10]. Those who reach out to support and help the victim of bullying are often referred to as a “defender.” Defenders in cyberbullying have long been viewed as those who provide help and support to victims, and defending behavior is a collection of helping strategies, i.e., defending behavior is viewed as a one-dimensional concept [11]. Alternatively, bystanders in cyberbullying have been classified into different types, such as reinforcer, assistant, defender, and outsider, based on the reactions that bystanders make after witnessing the bullying incident. For example, researchers have categorized bystanders in cyberbullying into different types, such as defender, assistant, reinforcer, and outsider [12,13,14]. This categorization has been extended to cyberbullying by drawing primarily on the types of bystander responses in school bullying (offline bullying) and following the types of divisions used in school bullying research.

This division by role rather than behavior can be problematic because someone may be excluded from other roles because they have similar scores in more than one role [11]. In other words, an individual’s role in a cyberbullying incident is dynamic, such that those who come forward to offer help may also be victims of cyberbullying. For example, cyberbullying victimization significantly and positively predicts cyberbullying perpetration, and those adolescents who are victims of cyberbullying are often the perpetrators of cyberbullying behaviors, who also take advantage of features such as the anonymity and ubiquity of the cyber environment to vent their emotions or seek revenge [15,16]. Therefore, in studies of cyberbullying defending behavior, the role division tends to confuse the types of defending behavior. For example, some researchers have argued that previous studies have not further distinguished between categories, such as positive and negative defending behaviors in cyberbullying [17]. In contrast, in cyberbullying, bystanders engage in a broad and diverse range of defending behaviors, and they use different strategies depending on the form of bullying. For example, some may provide emotional support (e.g., comforting the victims) and technical support (e.g., helping the victims block or delete information). Others will fight back with the bullied person, thus maintaining the bullied person’s safety or protecting the victim’s legal rights [18].

With the increasing research on cyberbullying, researchers mainly consider defending behavior in bullying incidents as a concept with a multidimensional structure rather than a collection of single strategies and try to differentiate the types of defending behavior [17]. For example, in a study in which researchers interviewed 113 adolescents to understand the bystander behavioral responses they endorsed during a cyberbullying incident, 16 different response types were identified, and bystander helping behaviors were categorized as direct defending and indirect defending. Direct defending refers to the response in the face of a cyberbullying incident and is directed toward the bully; indirect defending refers to providing help to the bullied by asking for help and reporting and is directed toward the bullied [19]. Another study also found that adolescents who witnessed cyberbullying were more likely to use direct defending if they perceived themselves as more powerful or capable than the bully. In contrast, adolescents who witnessed cyberbullying were more likely to use indirect defending if they perceived themselves as less capable of helping the victim or as having fewer resources available to them [20]. In addition to classifying defending behavior in cyberbullying into direct and indirect defending, some researchers have argued that a further distinction can be made between aggressive and prosocial defending, with aggressive defending representing a different type of defending in a different form. For example, bystanders will stand up for the victims and, in turn, fight back against the bully through verbal abuse, intimidation, and manhandling the bully [18]. Previous research has found that adolescents who witness cyberbullying incidents typically use aggressive behaviors to protect their bullied peers, such as verbally abusing the bully in retaliation or threatening the bully to stop bullying, in addition to using direct and indirect defending strategies [21].

Researchers use three main methods to measure and assess defending behavior. The first is the questionnaire method, in which researchers develop a cyberbullying bystander role scale or defending behavior scale that lists typical helpful bystander behaviors and guides study participants to respond according to their reactions to cyberbullying incidents. For example, in one study, researchers used a 50-question cyberbullying role scale corresponding to five roles (bully, bullied, defender, assistant, and outsider) [22]. In another study, a researcher presented 14 questions about general defending behaviors, and the research subjects made choices based on the descriptions provided in the questions [17]. The second method is the experimental method, in which the researcher establishes an experimental context to assess the behavioral responses of research subjects in the context of a cyberbullying incident. For example, researchers have set up cyberbullying situations in experiments where research subjects are guided to make choices in order to determine whether they are passive bystanders or active defenders in cyberbullying situations [23]. The third method is the interview method, in which the researcher engages in verbal discourse with the research subjects to ascertain the defending behaviors they have engaged in about cyberbullying, thereby determining their type of defending. For example, one researcher conducted in-depth interviews with study participants to gain insight into their defending strategies during cyberbullying incidents [19].

Of these three methods, the questionnaire method is the most widely used. Researchers have measured defending behavior by adapting or developing existing questionnaires. For example, the Styles of Bystander Intervention Scale developed by Moxey and Bussey consists of two dimensions: aggressive intervention (e.g., “making threats to the bully”) and constructive intervention (e.g., “Encourage the child to report that he or she being bullied”), respectively [24]. Overall, the previous scale was deficient in two ways: First, the items were mostly directly adapted from school bullying (offline bullying). The second deficiency was the simplification of bystander behavior and the use of a simplified questionnaire for the measurement of defending behaviors in cyberbullying. These shortcomings cause the research on defending behavior to face the problem of inaccurate measurement and incomplete description.

In sum, the defending strategies employed by adolescents in cyberbullying are diverse and multifaceted, extending beyond the scope of a mere aggregation of discrete behaviors as delineated and evaluated by some bystander role scales. Furthermore, most extant studies must clarify the various forms of defending actions, failing to make additional distinctions. In conjunction with the attributes of the online milieu, including anonymity and a vast potential audience, the defending strategies employed by adolescents in response to cyberbullying incidents diverge significantly from those observed in offline bullying. For example, the defending behaviors exhibited by bystanders in the context of offline bullying are more costly and risky. In contrast, those observed in the context of cyberbullying are less costly and less risky. In light of the above, the primary objective of this study is to examine the structure and dimensions of defending behavior in cyberbullying and to develop a scale that is appropriate for measuring defensive behavior in cyberbullying among adolescents (aged 13–19 years), which can provide a foundation for a more comprehensive and more in-depth study of defending behavior in cyberbullying among this age group.

## 2. The Present Study

Defending behavior in the context of cyberbullying can be defined as a type of prosocial behavior adopted by bystanders to prevent the exacerbation or worsening of cyberbullying incidents. Consequently, investigating the structure and dimensions of defending behavior in cyberbullying can provide a foundation for a comprehensive examination of prosocial behavior in cyberbullying research. In this study, adolescents who have provided assistance and support to victims of cyberbullying were selected for in-depth interviews. The objective was to gain insight into the connotations of defending behavior and identify preliminary types of defending behavior. Following this, a psychometrically compliant scale on defending behavior was developed. Subsequently, the reliability and validity of the scale were validated in a group of Chinese adolescents. Finally, we also tested the measurement equivalence of the scale and analyzed gender differences.

## 3. Methods

Following the principles of scale development by Boateng and colleagues [25], we developed and validated the defending behavior scale of cyberbullying for adolescents in several key phases. Phase 1 consisted of dimension identification and item development. We first clarified the concept and dimensions through a qualitative study of in-depth interviews. Combining the results of the qualitative analysis and drawing on previous scales, items were generated. Phase 2 consisted of scale development and factor extraction. We constructed the scale from questionnaires to reduce the items and to evaluate factor structures. Phase 3 consisted of scale evaluation and validation. We assessed the scale by testing reliability, validity, and invariance across gender groups. In addition, the sample size was an essential issue in the scale development and validation process. Based on the development and best practices for validating behavioral scales, we used three samples throughout the validation process and collected data from different samples [25]. Researchers have suggested that a sufficiently large sample should be used to assess the psychometric properties of scale scores. A smaller sample size of approximately 250~350 should be used if the study population includes patients, and a larger sample size of approximately 500~600 should be used if the study population includes students [26]. Therefore, in this study, we used three different samples of adolescents. Sample 1 (*n* = 20) was used for qualitative analysis. Sample 2 (*n* = 550) was used as an exploratory factor analysis subject. After the scale items were adjusted based on the results obtained, Sample 3 (*n* = 526) was used as a validation factor analysis subject to obtaining a formal version of the scale.

### 3.1. Phase 1: Dimension Identification and Item Development

#### 3.1.1. Sample 1

Purposive sampling was used to select the interviewees. The number of interviewees was based on the principle of saturation of interview data, and 20 adolescents from a middle school were selected as interviewees. The inclusion criteria for the interviewees were as follows: aged 13~18 years old; voluntary participation in the study and signing of an informed consent form; and having experienced cyberbullying and having helped the victim during the incident. A total of 20 adolescents were interviewed for this study, including 9 boys and 11 girls, with a mean age of 16.60 years (SD = 1.05). Prior to the formal interviews, the participants completed the cyberbullying bystander behavior scale [27]. The scale was divided into three subscales, namely, defender, outsider, and assistant, and was scored on a 5-point Likert scale. The mean score of the interview subjects on the defender subscale was 4.13 (SD = 0.57), considerably higher than the theoretical mean of the scale (M = 2.5). This finding indicates that the interviewed students exhibited defending behaviors in cyberbullying.

#### 3.1.2. Dimension Identification

Based on the types and characteristics of helping behaviors found in previous research [19,20], in-depth interviews were conducted with Sample 1. The main content of the interviews included basic information about the subjects (e.g., gender, age); essential cell phone use (e.g., number of years of use, experience of use); understanding of cyberbullying defending behaviors and views on specific strategies; experiences regarding defending behaviors related to cyberbullying; feelings and thoughts when witnessing cyberbullying; and the defending strategies adopted by the interviewees and their effects.

Before the formal interview, we gave the interviewees a detailed explanation of the purpose of the study. In order to help the interviewees better understand cyberbullying, we presented typical cases to them in advance, which was carried out to ensure that the interviewees had some knowledge and understanding of the concepts and forms of cyberbullying. The interviewees signed an informed consent form after knowing and agreeing to it. With the consent of the interviewees, we audio-recorded the entire interview.

After obtaining the subjects’ consent, we transcribed the interviews from the audio recordings to text. First, all textual information was coded by content analysis, with declarative sentences as the unit of analysis, and a total of 51 declarative sentences were obtained. Then, the declarative sentences were sorted out, and 10 sentences unrelated to defending behaviors and semantically ambiguous sentences were deleted, which was followed by merging 21 declarative sentences with similar contents. Again, the declarative sentences were merged at the conceptual level, and these sentences were categorized. Finally, 20 declarative sentences about defending behaviors were compiled into four categories: comforting and accompanying the victim, reporting to an authority figure, counterattacking and retaliating against the bully, and figuring out how to solve the problem (see Table 1).

#### 3.1.3. Item Construction

The items of the initial scale were constructed in three ways: the first was by reviewing the qualitative analysis of the original data and summarizing from the self-reporting of the interviewees to obtain the topics, for example, “I sent a message to the victim to encourage him/her”; the second was to build based on the original data, and based on the four dimensions constructed by the content analysis, we compiled some questions, such as, “I sent a message to show concern to the victim of cyberbullying” and “I reported the cyberbullying behavior to the webmaster”; the third was to refer to some questions from previous scales [28,29,30,31,32], such as, “I told a teacher about the cyberbullying incident”. When the questionnaire items were in English, they were translated into Chinese by the first author and reviewed by a bilingual (Chinese and English) expert.

### 3.2. Phase 2: Scale Development and Factor Extraction

#### 3.2.1. Sample 2

For the initial evaluation of items, after obtaining approval for this study from the ethics committee of the authors’ institution, a convenience sampling method was employed to select 600 students attending two middle schools in Sichuan Province as study participants. After obtaining informed consent from those involved (headmasters, teachers, and students), the students completed self-report questionnaires during their mental health education course in the presence of a researcher and teachers. Prior to the commencement of the survey, the researcher emphasized to the students the principles of anonymity and the confidentiality of this study. In addition, the researcher informed all participating students that they could refuse to participate in the study or withdraw from the study at any time if they wished to do so without penalty and that the content of the questionnaires they completed would not be disclosed to anyone. All participating students completed the paper questionnaire during class. A total of 550 valid questionnaires were collected for analysis, representing a 91.67% recovery rate. Of the participants, 241 (43.8%) were male and 309 (56.2%) were female. The initial subjects ranged in age from 13 to 19 years, with a mean age of 16.44 years (SD = 1.07).

#### 3.2.2. Scale Development

An initial scale was developed based on the above approach. We asked one psychology professor, two doctoral students majoring in psychology, and one doctoral student majoring in Chinese to examine the reasonableness of the item setting and the fluency of language expression, and we deleted semantically ambiguous or inappropriate items and finally retained 34 items as the items of the initial scale. The initial scale consisted of 34 items and was scored on a 5-point Likert scale, with 1 representing “completely disagree”, 2 representing “disagree”, 3 representing “not sure”, 4 representing “agree”, and 5 representing ‘completely agree’.

The data from Sample 2 were itemized. We calculated the total scores of the participants on the items of the initial scale, followed by selecting the top 27% of the participants with the highest scores on the total scores of the initial scale as the high group and the bottom 27% of the participants with the lowest scores on the total scores of the initial scale as the low group, and we then analyzed the difference between the means of each item in the high group and the low group by using the independent samples *t*-test. The test value of the difference between the means of the high and low subgroups was obtained, which is the decision value of the item differentiation (CR value). If the decision value reaches the significance level, the item discriminates, and if not, the item does not discriminate and should be considered for deletion.

#### 3.2.3. Factor Extraction

We used SPSS 21 to perform an exploratory factor analysis on the data from Sample 2, and we first performed the Kaiser–Meyer–Olkin (KMO) test and Bartlett’s sphericity test on the data. The KMO value ranges from 0 and 1, and if it is closer to 1 and the results of Bartlett’s sphericity test reaches the significance level, the data are suitable for exploratory factor analysis. Then, we extracted the factors using principal component analysis, rotated the factors by the maximum variance method, and extracted the eigenvalues. Based on the results of the factor analysis, the dimensions of the initial scale and the names of the dimensions were interpreted.

### 3.3. Phase 3: Scale Evaluation and Validation

#### 3.3.1. Sample 3

For reliability, validity analyses and measurement equivalence testing were conducted. Participants were recruited using a convenience sampling method after obtaining approval for this study from the ethics committee of the authors’ institution. Two secondary schools in Chongqing Municipality were selected for the study. After obtaining informed consent from stakeholders (principal, teachers, and students), the students completed self-report questionnaires in the presence of a researcher and a teacher during a mental health education course. Before the start of the survey, the researcher emphasized the anonymity and confidentiality of this study to the students. Furthermore, the researcher informed all participants that they could refuse to participate in the study or withdraw from the study at any time if they wished without any penalty and that the content of the questionnaires they completed would not be disclosed to anyone. All participants completed the paper questionnaire during the course, and no student refused or withdrew from this study. A total of 526 valid questionnaires were collected, representing a recovery rate of 87.67%. Of the participants, 203 (38.6%) were male and 323 (61.4%) were female. The subjects’ ages ranged from 13 to 19 years, with a mean age of 16.73 (SD = 1.06).

#### 3.3.2. Measures

Prior research has indicated that adolescent cyberbullying behavior is not significantly or significantly negatively correlated with defending behavior in cyberbullying [9]. The intention of adolescents to intervene in cyberbullying was found to be significantly and positively correlated with their defending behavior [33]. Accordingly, this study employed the Cyberbullying Scale and the Intention to Help Cyberbullying Victims Scale as criterion validity scales to assess the validity of the scale associations of the developed scale.

The Cyberbullying Scale, developed by Patchin et al., was utilized to assess adolescent cyberbullying behavior [34]. The scale is unidimensional in structure and encompasses eight discrete cyberbullying behaviors (e.g., “I threatened to hurt someone online”). The scale employs a 5-point Likert scale, with options ranging from “never” to “many times” and a scoring range of 1 to 5. Higher scores indicate a higher frequency of cyberbullying behavior. The internal consistency coefficient of the scale was 0.86 in the present study.

The intention to intervene in cyberbullying among adolescents was assessed using the Intention to Help Cyberbullying Victims Scale, developed by Hayashi and Tahmasbi [35]. This scale comprises three items (e.g., “If I saw someone being bullied online, I would help that person”). The scale is scored on a 7-point Likert scale, with options ranging from “completely disagree” to “completely agree.” The scores of the three items were added together to obtain the final total. Higher scores indicate a stronger intention to intervene. The internal consistency coefficient of the scale was 0.81 in the present study.

#### 3.3.3. Tests of Reliability and Validity

The validity analysis included structural validity, convergent validity, discriminant validity, and criterion validity. We used Mplus 8.3 to analyze the data for structural validity. If χ^2^/df < 5, RMSEA < 0.08, SRMR < 0.08, CFI > 0.90, and TLI > 0.90, the structural validity of the compiled scale was good. If the factor loadings of the items corresponding to the dimensions of the scale were all above 0.6, each latent variable corresponded to the topic to which it belonged with good representativeness; if the average variance extracted (AVE) for each dimension was greater than 0.5 and the composite reliability (CR) was greater than 0.8, the convergent validity of the model was more satisfactory. In addition, if the correlation coefficients among the scale dimensions were less than the square root of the corresponding AVE, there was some correlation among the dimensions of defending behavior. In order to test the criterion validity of the scale, we used SPSS 21.0 to analyze the relationship between adolescent defending behavior and cyberbullying behavior and intervention intention in cyberbullying. Finally, to test the measurement equivalence of the scale across genders, we used Mplus 8.3 to test the data from Sample 3 for gender measurement equivalence. Gender differences were then be analyzed using independent samples *t*-tests.

## 4. Results

### 4.1. Item Analysis

The total scores of the study participants on the 34 items of the initial scale were calculated, and then the top 27% of the participants who scored the highest on the total scores of the initial scale were selected as the high group, and the bottom 27% of the study participants who scored the lowest on the total scores of the initial scale were selected as the low group. Then, the test of the difference between the scores of the high and low subgroups on each question item was conducted, and finally, the test value of the difference between the means of the high and low subgroups, which is the decision value of the item differentiation (CR value), was obtained. The results showed that the decision value of each item of the scale reached the significance level, and this result indicates that the 34 items of the original scale have a reasonable degree of differentiation.

### 4.2. Exploratory Factor Analysis

Firstly, the data obtained from the scale were subjected to the Kaiser–Meyer–Olkin (KMO) test and Bartlett’s test of sphericity. In this study, the value of KMO was 0.93, and the Bartlett’s test of sphericity chi-square value was 12,127.30, with a degree of freedom of 561, and with *p* < 0.001, indicating that the data were suitable for exploratory factor analysis.

Secondly, the factors were extracted using principal component analysis and rotated by the maximum variance method. This resulted in the extraction of four factors with eigenvalues greater than 1, which collectively explained 63.41% of the total variance. Furthermore, the pertinent items were excluded in accordance with the following criteria: (1) the common degree was less than 0.4; (2) the factor loadings were less than 0.4; (3) and there were double loadings, and the double loadings were all above 0.4, with a difference of less than 0.3 between them. Based on the criteria above, 10 items were removed from the preliminary test scale. At this juncture, the preliminary test scale comprised 24 items. Four factors with eigenvalues exceeding one were identified, collectively explaining 66.26% of the total variance. The factor loadings, factor eigenvalues, and contribution rates for each item are presented in Table 2.

Finally, the four factors were analyzed and named according to the meanings of the items they contained: the first factor was named “emotional support”, which describes focusing on the emotions of the victim, where the bystander understands, comforts, and accompanies the victim during a cyberbullying incident. The second factor was named “reporting authority”, which describes focusing on outside resources, where bystanders report incidents of cyberbullying to adults who can offer help and support. The third factor, named “aggressive defending”, describes focusing on the bullies, where bystanders verbally abuse or threaten cyberbullies. The fourth factor, named “problem-solving”, describes focusing on the current cyberbullying incident and independently working to figure out a solution to the problem or situation. The final scale can be found in Appendix A.

### 4.3. Reliability Analysis

Reliability analysis was conducted on the defending behavior scale of cyberbullying for adolescents. The results demonstrated that the internal consistency coefficient of the scale was 0.92, while the internal consistency coefficients of the dimensions of emotional support, reporting authority, aggressive defending, and problem-solving were 0.88, 0.92, 0.92, and 0.87, respectively.

### 4.4. Validity Analysis

#### 4.4.1. Structural Validity

The structural validity of the scale was subjected to analysis. A factor analysis was conducted using Mplus 8.3 to test the structural validity of the scale, with the four-factor structure being validated. The model fit indices were χ^2^/df = 4.068, RMSEA = 0.076, SRMR = 0.053, CFI = 0.912, and TLI = 0.902. The results indicated that the four-factor structural model fit had good structural validity.

This study compared competing models to validate the four-factor model as the optimal model. First, defending behavior was viewed as a single-factor structure. Second, according to the findings of previous studies, defending behavior can be divided into direct defending, which refers to a response in the face of a bullying incident that is directed at the bully, with typical strategies such as fighting back against the bully, and indirect defending, which refers to the provision of help to the bullied by asking for help and reporting, pointing to the target of the bullied, such as reporting bullying to an adult [19,35]. Previous research has also found that adolescents who witness cyberbullying incidents often use aggressive behaviors to protect the bullied [21]. Therefore, a one-factor model; a two-factor model, i.e., direct defending (aggressive defending + problem-solving) and indirect defending (emotional support + reporting authority); a three-factor model, i.e., direct defending (problem-solving), indirect defending (emotional support + reporting authority), and aggressive defending; and a four-factor model, i.e., emotional support, reporting authority, aggressive defending, and problem-solving, were developed. Comparing the above model fitting indices, it can be seen that the four-factor structural model, where χ^2^/df = 4.068, which is less than 5; CFI = 0.912, TLI = 0.902, which are both greater than 0.9; and RMSEA = 0.076, SRMR = 0.053, which are both less than 0.08, had a more desirable model structure (see Table 3).

#### 4.4.2. Convergent Validity

We analyzed the convergent validity of the scale. The results demonstrated that the factor loadings of the four dimensions of the scale corresponding to the question items were all above 0.6, indicating that each latent variable corresponding to the topic to which it belongs was adequately represented. Furthermore, the average variance extracted (AVE) for each dimension was greater than 0.5, and the composite reliability (CR) was greater than 0.8, indicating that the convergent validity of the model was more satisfactory.

#### 4.4.3. Discriminant Validity

The discriminant validity of the scale was analyzed. As illustrated in Table 4, the correlation coefficients between the four dimensions of the scale were less than the square root of the corresponding AVE, indicating that although the four dimensions were correlated, they also exhibited some degree of differentiation, thereby demonstrating good discriminant validity.

#### 4.4.4. Criterion Validity

We also analyzed the criterion validity of the scale. As illustrated in Table 5, the correlation between the dimensions of the scale ranged from −0.09 to 021. The results demonstrated that emotional support was not significantly correlated with cyberbullying, while reporting authority was negatively correlated to a significant extent, aggressive defending was positively correlated, and problem-solving was insignificantly correlated. Furthermore, all scale dimensions were significantly and positively correlated with the bystander’s intention to help, with correlation coefficients ranging from 0.22 to 0.71. Defending behavior was also significantly and positively correlated with the bystander’s intention to help, with a correlation coefficient 0.76.

### 4.5. Gender Difference

First, we performed a measurement equivalency test on the data from Sample 3. The study constructed four models, of which the configural invariance (M1) allowed for all parameters to be estimated freely; the metric invariance (M2) set the factor loadings to be equal for both male and female; the scalar invariance (M3) was used to set the intercepts further to be equal on top of M2; and the strict invariance (M4) added residuals to be equal on top of M3 [36]. If ΔCFI and ΔTLI are less than 0.10 in the comparison of M2 with M1, M3 with M2, and M4 with M3, the scale is consistent across genders [37]. The equivalence test found that the model indicators were well fitted, and ΔCFI and ΔTLI were all less than 0.10 in the comparisons of M2 with M1, M3 with M2, and M4 with M3. The differences in the model fit metrics and fit indices for each equivalent model are shown in Table 6.

Second, we tested for differences between male and female students. As shown in Table 7, male students scored significantly higher than female students on both aggressive defending (*p* < 0.001) and defending behavior (*p* < 0.01).

## 5. Discussion

This study developed the defending behavior scale of cyberbullying for adolescents. The scale exhibited satisfactory psychometric properties and is a reliable and valid measurement tool for assessing defending behavior. The initial clarification of the connotation and structure of adolescents’ defending behavior was achieved through the use of in-depth interviews. The scale was developed for the adolescent group through exploratory and validation factor analysis, resulting in a reliable and valid instrument. The scale comprises four dimensions: emotional support, reporting authority, aggressive defending, and problem-solving. The internal consistency coefficient for the scale was 0.92, and the internal consistency coefficients for the four dimensions were 0.88, 0.92, 0.92, and 0.87, respectively. The reliability analysis results showed that the developed defending behavior scale had high reliability. The results of the validated factor analysis showed that the χ^2^/df value of the four-factor model was less than 5, the values of CFI and TLI were above 0.90, the RMSEA was less than 0.08, and the fit indices of the indicators were better, which indicated that the scale had a good structural validity. The convergent validity analysis revealed that the factor loadings of the items corresponding to the four dimensions of the scale were all greater than 0.5, indicating that each dimension was represented by the question to which it belongs, with good representativeness. The discriminant validity of the four scale dimensions was evaluated, and the findings indicated that the four dimensions exhibited a certain degree of correlation and a sufficient level of differentiation, thereby supporting the conclusion that the scale demonstrated good discriminant validity.

We also conducted a criterion-related validity analysis of the scale. The results indicated that the correlations between the dimensions of the scale and cyberbullying behavior were non-significant or significantly negative, with a significant positive correlation between the aggressive defending dimension and cyberbullying behavior, which suggests that individuals who use aggressive defending in cyberbullying are also more inclined to bully others in cyberbullying incidents, a result that is also in line with the findings of previous studies [12]. In addition, there was a significant positive correlation between the total score and dimensions of the defending behavior scale and bystander’s intention to help. The findings were also consistent with previous research [33], suggesting that the stronger the adolescents’ intention to make interventions in cyberbullying, the higher the likelihood that they will engage in defending behaviors in actual cyberbullying incidents.

We identified four types of defending: emotional support, reporting authority, aggressive defending, and problem-solving. Previous research has also distinguished between types of defending behavior in cyberbullying [17]. For example, adolescents who witness cyberbullying are more likely to resort to direct defending when they perceive themselves as more powerful or capable than the bully. Direct defending refers to responses directed toward the bully when confronted with a bullying incident. Typically, direct defending includes fighting back against the bully, consistent with the aggressive defending in this study.

In contrast, adolescents who witness cyberbullying incidents are more likely to use indirect defending when they perceive their ability to help the victim to be low or have few resources available to them [38]. This result is generally consistent with the reported authority found in this study. In addition, some researchers have identified aggressive and prosocial types of defending, with aggressive defending representing a different form of another type of defending, such as bystanders who stand up for the bullied and, in turn, strike back at the bully in the form of verbal abuse, intimidation, and mansplaining [18]. Some studies support this view, suggesting that adolescents who witness cyberbullying may use aggressive behaviors to defend their bullied peers, such as verbally abusing the bully in retaliation or threatening the bully to stop the bullying behavior, in addition to direct and indirect defending strategies [21]. The aggressive defending found in this study is consistent with the results of previous studies.

The four types found in this study are mainly consistent with offline bullying. Previous studies have found that Chinese students use tactical defending, a strategy specific to offline bullying [28]. We did not find this strategy, which may be due to the anonymity characteristic of the online environment. It makes it less likely for bystanders to fail to notice when they engage in defending behaviors and, therefore, less likely to be concerned. However, because of the unique environment of cyberbullying (e.g., anonymity), we also found that reporting incidents of cyberbullying to an authority was a commonly used strategy.

Finally, this study also found differences in defending behavior between boys and girls. At the overall level, there is a clear gender difference in adolescents’ defending behaviors in cyberbullying, as shown by the fact that boys will engage in more defending behaviors. Also, boys will be more likely to employ aggressive defending. Previous research on gender differences in defending behavior has been controversial. For example, girls usually provide emotional help during cyberbullying incidents, such as comforting and accompanying the bullied [11]. In contrast, boys take a more direct approach, such as joining in with the victim to fight back, e.g., by threatening or verbally abusing the bully [39]. However, it has also been found that there are no significant gender differences in defending behaviors between boys and girls and that both boys and girls provide help to the bullied when confronted with cyberbullying incidents [6].

The results of this study also have some implications and limitations. First, defending behavior is a typical prosocial behavior, and most of the data in this study came from self-reported questionnaires, which makes them susceptible to social expectations. Second, only secondary school students in two regions of China were selected for our study. In the course of our study, we found that the schools had strict regulations on secondary students’ use of cell phones to access the internet, so their experience with the internet and even the amount of time they spend using the internet may have had an impact, which may limit the generalizability of the findings. Future research could select adolescents from different cultural backgrounds as study subjects and explore whether they differ in their strategies.

## 6. Conclusions

The defending behavior scale in cyberbullying for adolescents, developed in this study, comprises 24 items that assess four dimensions: emotional support, reporting authority, aggressive defending, and problem-solving. The scale demonstrates good reliability and validity and can be utilized to assess adolescents’ defending behaviors in cyberbullying.

## Figures and Tables

**Table 1:** Results of qualitative analysis.

Dimension	Dimension Description	Representative Item
Comforting and accompanying the victim	Comforting and accompanying reflects the emotional comfort and support provided by individuals to victim of cyberbullying.	I send messages to the victim to encourage him/her.
Reporting to an authority figure	The individual reports the cyberbullying incident to an adult who can resolve it, such as a teacher or network administrator.	I reported the cyberbullying to the webmaster.
Counterattacking and retaliating against the bully	Counter retaliation is manifested when an individual strikes back at the issuer of cyberbullying by threatening or abusing them to force them to remove or withdraw relevant information or content.	I sent a message verbally abusing the bully to stop the cyberbullying behavior.
Figuring out how to solve the problem	Individuals focus their attention on the cyberbullying incident and independently work on their own to figure out a solution to the problem or situation that the bullied person is experiencing in order to stop the bullying from worsening.	I help cyberbullied people respond to untrue comments from others.

**Table 2:** Exploratory factor analysis.

Item	Factor 1	Factor 2	Factor 3	Factor 4	Communality
Item 24	0.870				0.762
Item 20	0.860				0.752
Item 23	0.839				0.708
Item 19	0.837				0.704
Item 22	0.818				0.690
Item 21	0.789				0.659
Item 18	0.724				0.551
Item 16		0.858			0.817
Item 13		0.829			0.722
Item 17		0.828			0.776
Item 11		0.776			0.652
Item 15		0.690			0.602
Item 12		0.666			0.574
Item 1			0.803		0.701
Item 5			0.765		0.681
Item 4			0.715		0.565
Item 2			0.702		0.544
Item 6			0.679		0.630
Item 8			0.641		0.549
Item 28				0.785	0.768
Item 27				0.765	0.748
Item 29				0.731	0.619
Item 30				0.714	0.623
Item 34				0.657	0.504
Eigenvalue	7.719	4.772	1.891	1.519	
Contribution Rate %	32.161	19.883	7.881	6.330	
Cumulative Contribution Rate %	32.161	52.044	59.925	66.255	

Note: factor 1 = aggressive defending; factor 2 = reporting authority, factor 3 = emotional support, factor 4 = problem-solving.

**Table 3:** Validated factor analysis model fit index.

Model	χ^2^/df	RMSEA	CFI	TLI	SRMR
One-factor model	11.680	0.142	0.534	0.490	0.167
Two-factor model	9.080	0.124	0.649	0.614	0.209
Three-factor model	4.678	0.084	0.842	0.824	0.065
Four-factor model	4.068	0.076	0.912	0.902	0.053

**Table 4:** Distinguishing validity of dimensions.

	CR	AVE	Emotional Support	Reporting Authority	Aggressive Defending	Problem-Solving
Emotional support	0.88	0.56	—			
Reporting authority	0.92	0.65	0.70	—		
Aggressive defending	0.92	0.62	0.09	0.08	—	
Problem-solving	0.87	0.58	0.69	0.74	0.18	—
Square root value of AVE			0.75	0.81	0.79	0.76

**Table 5:** Criterion-related validity analysis.

	Cyberbullying	Bystander’s Intention to Help
Emotional support	0.10	0.65 ***
Reporting authority	−0.09 *	0.69 ***
Aggressive defending	0.21 ***	0.22 ***
Problem-solving	−0.03	0.71 ***
Defending behavior	0.03	0.76 ***

Note: * *p* < 0.05, *** *p* < 0.001.

**Table 6:** Fit indicators for equivalent models (N = 526).

Model	S-Bχ^2^	df	CFI	TLI	RMSEA [90% CI]	SRMR	ΔCFI	ΔTLI
M1	1060.829	492	0.915	0.905	0.066 [0.061, 0.072]	0.061		
M2	1083.030	512	0.915	0.908	0.065 [0.060, 0.071]	0.063	0.000	0.003
M3	1115.988	532	0.913	0.910	0.065 [0.059, 0.070]	0.064	−0.002	0.002
M4	1152.396	556	0.911	0.912	0.064 [0.059, 0.069]	0.065	−0.002	0.002

Note: M1 = configural invariance, M2 = metric invariance, M3 = scalar invariance, M4 = strict invariance.

**Table 7:** Gender comparison of scores in adolescent populations (M ± SD).

	Male (N = 203)	Female (N = 323)	t	*p*
Emotional support	20.99 ± 4.20	20.26 ± 4.85	1.76	0.078
Reporting authority	19.95 ± 4.83	19.50 ± 4.96	1.03	0.304
Aggressive defending	14.61 ± 5.25	12.72 ± 4.87	4.20	0.000
Problem-solving	16.84 ± 3.81	16.68 ± 4.01	0.44	0.657
Defending behavior	72.37 ± 12.32	69.15 ± 14.10	2.68	0.008

## Data Availability

The data presented in this study are available on request from the corresponding author.

## Appendix A

When we use social media, such as QQ, WeChat, Weibo, and Baidu Post Bar, people often express their malice towards others and even attack them, such as insulting, ostracizing, and harassing through the Internet. These bullying behaviors that occur online are called cyberbullying. We refer to the person who harms as the bully and the person who suffers harm from bullying as the bullied. The following questions are about what you did when you witnessed someone being bullied online sometime in the past. Please read the following questions carefully, determine their consistency with your experience, and tick the appropriate option.

1. I sent or left a message to comfort the bullied person.
2. I told the bullied person that it was not their fault for being bullied.
3. I tried to cheer up the bullied person.
4. I sent or left a message to show concern for the bullied person.
5. I gave the bullied person some advice (e.g., how to get help).
6. I took the time to listen to the bullied person.
7. I told the teacher about the cyberbullying.
8. I reported the cyberbullying to the webmaster.
9. I told my parents about the cyberbullying.
10. I told an adult capable of resolving the cyberbullying.
11. I told my teacher about an incident of cyberbullying.
12. I figured out how to get an administrator to handle it after the cyberbullying.
13. I retaliated against the bully.
14. I sent an abusive message to the bully to stop the cyberbullying.
15. I ridiculed the bully and stood up for the victim.
16. I asked classmates and friends to join me in fighting against the bully.
17. I said terrible things about the bully.
18. I threatened the bully.
19. I verbally abused the bully.
20. I persuaded the bully or others involved to stop cyberbullying.
21. I cautioned the bully that their behavior was wrong and they should stop immediately.
22. I helped the bullied person respond to untrue comments made by others.
23. I encouraged the bully to apologize to the bullied person.
24. I told people that the bullied person is innocent.
